# Temperature enhances the functional diversity of dissolved organic matter utilization by coastal marine bacteria

**DOI:** 10.1111/1758-2229.13123

**Published:** 2022-09-14

**Authors:** Xosé Anxelu G. Morán, Nestor Arandia‐Gorostidi, Tamara Megan Huete‐Stauffer, Laura Alonso‐Sáez

**Affiliations:** ^1^ Centro Oceanográfico de Gijón/Xixón IEO‐CSIC Gijón/Xixón Spain; ^2^ Department of Marine Biology and Oceanography Institute of Marine Sciences, CSIC Barcelona Spain; ^3^ AZTI, Marine Research, Basque Research and Technology Alliance (BRTA) Sukarrieta Spain

## Abstract

Although bulk bacterial metabolism in response to temperature has been determined for different oceanic regions, the impact of temperature on the functional diversity of dissolved organic matter (DOM) utilization has been largely unexplored. Here, we hypothesized that besides modifying the rates of carbon utilization, temperature can also alter the diversity of substrates utilized. The patterns of utilization of 31 model DOM compounds (as represented in Biolog EcoPlate™) by bacterioplankton were assessed using inocula from surface waters of the southern Bay of Biscay continental shelf over 1 year. Bacteria utilized more polymers and carbohydrates in late spring and summer than in winter, likely reflecting changes in substrate availability linked to the release and accumulation of DOM in phytoplankton post‐bloom conditions. Seawater temperature correlated positively with the number of substrates utilized (i.e. functional richness) and this relationship was maintained in monthly experimental incubations spanning 3°C below and above in situ values. The enhancement of functional richness with experimental warming displayed a unimodal response to ambient temperature, peaking at 16°C. This temperature acted as a threshold separating nutrient‐sufficient from nutrient‐deficient conditions at the study site, suggesting that trophic conditions will be critical in the response of microbial DOM utilization to future warming.

## INTRODUCTION

Heterotrophic bacteria and archaea play a key role in the remineralization of dissolved organic matter (DOM) in the ocean. The myriad DOM compounds both produced in situ (ultimately derived from phytoplankton primary production) and advected from elsewhere (e.g. terrestrial inputs) have resulted in specialized niches exploited by a huge bacterial diversity (Nelson & Wear, 2014). Molecular studies have unveiled a large variety of enzymes produced by marine bacteria to access and utilize DOM (e.g. see references in the study by Arnosti et al., 2021) and membrane transporters used to incorporate carbon monomers inside the cells (Bergauer et al., 2018; Poretsky et al., 2010). Yet, the diversity of DOM substrates potentially processed by heterotrophic bacteria is usually overlooked (Moran et al., 2016). One simple way of addressing the specialization of environmental assemblages in carbon utilization is through using commercial kits containing multiple biodegradable carbon sources, such as Biolog EcoPlate™. In this assay, the potential functional diversity of marine heterotrophic bacteria can be approached by the number of substrates (out of 31) being utilized in a short period (typically days). This method has been widely used with samples from different environments, from soils and wastewater treatment plants to natural aquatic samples. Despite its caveats (Preston‐Mafham et al., 2002), it has successfully addressed the differences in functional diversity along temporal and spatial marine and freshwater gradients (Christian & Lind, 2006; Ruiz‐González et al., 2015; Sala et al., 2005; Sala et al., 2008).

In the context of global change, temperature emerges as one of the most important environmental drivers of microbial plankton structure and function (Sunagawa et al., 2015). Warming has been shown to enhance both bulk microbial DOM transformation (Lønborg et al., 2020) and extracellular enzymatic activity rates (Baltar et al., 2017) of heterotrophic bacteria when nutrients are sufficient, which may result in larger standing stocks especially in mid‐ and high‐latitude regions (Morán et al., 2015, 2017). Also, temperature may induce qualitative changes in the array of DOM substrates being used, selecting for different types of compounds in cool and warm conditions (Grover & Chrzanowski, 2000). However, to our knowledge, no systematic evaluation of the role of temperature on the physiological profiling of bacterial communities using Biolog EcoPlate™ has yet been attempted. Whether only rates or also patterns of DOM utilization should be affected by temperature, remains unclear (Christian & Lind, 2006).

In this study, we inoculated Biolog EcoPlate™ with natural bacterioplankton assemblages from a temperate coastal site described in detail elsewhere (Alonso‐Sáez et al., 2015; Alonso‐Sáez et al., 2020) during a full year, combining in situ and experimental conditions. Annually recurrent dynamics in heterotrophic bacterial abundance (Morán et al., 2015), taxonomic diversity (Alonso‐Sáez et al., 2015; García et al., 2015) and growth rates (Huete‐Stauffer et al., 2015) have been previously reported at the study site, together with the temperature response of microbial plankton in terms of growth rates and standing stocks (Arandia‐Gorostidi et al., 2017; Huete‐Stauffer et al., 2015; Morán et al., 2018). Here, we aimed at testing the impact of temperature on the spectrum of utilizable carbon substrates or functional richness along seasonally changing conditions. Our hypotheses were that: (i) patterns of carbon source utilization vary according to trophic state and (ii) warming consistently modulates functional richness along the year.

## EXPERIMENTAL PROCEDURES

During 2012, we took monthly samples of seawater at the surface (ca. 5 m) of our reference station G2, with 110 m depth, located on the continental shelf of the northern Iberian Peninsula off Gijón/Xixón (Huete‐Stauffer et al., 2015). No sampling was performed in April because of logistic problems, which we tried to circumvent by taking two separate samples in May (5th and 23rd). Hereinafter, the former date will be referred to as ‘April’ and the latter one as ‘May’. The abundance, cellular characteristics and specific growth rates of heterotrophic bacterioplankton were determined by flow cytometry analysis (see Arandia‐Gorostidi et al., 2020; Huete‐Stauffer et al., 2015; Morán et al., 2018 for details), thus allowing the differentiation between low (LNA) and high (HNA) nucleic acid content cells (Morán et al., 2015). Ancillary physico‐chemical and biological variables such as stratification index (SI, the per metre difference in seawater density between 5 and 75 m depth), inorganic nutrient concentrations, phytoplankton biomass (using chlorophyll *a* concentration as a proxy) and dynamics were measured as detailed in the aforementioned studies. Briefly, two sets of triplicate 2 L samples, one unfiltered and the other one pre‐filtered through 0.8 μm, were incubated in parallel, mimicking in situ conditions. Daily changes in chlorophyll *a* in the unfiltered samples were used to estimate phytoplankton net growth rates (Morán et al., 2018) while heterotrophic bacterioplankton abundance in the pre‐filtered samples was monitored one to two times per day by flow cytometry in order to estimate their specific growth rates (Huete‐Stauffer et al., 2015). Both calculations were done as the slope of linear regressions of natural logarithm‐transformed standing stocks versus time in days.

**FIGURE 1:**
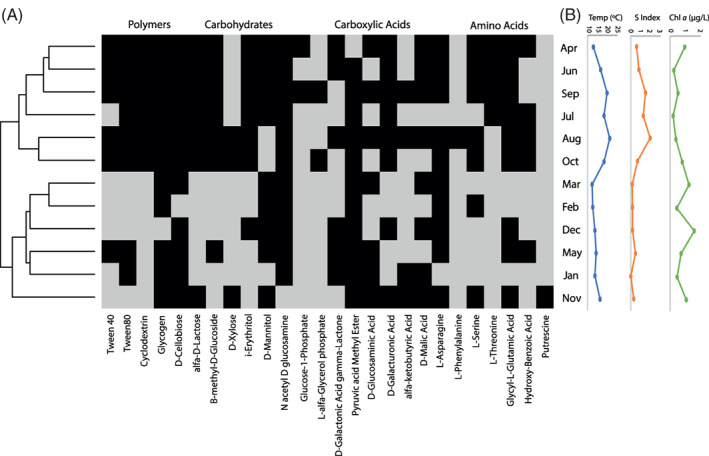
(A) Heatmap (black, positive; grey, negative) and clustering of individual carbon sources of biolog EcoPlate™ at in situ temperature in the 12 samples collected in 2012 from the surface of the study site (2 positive wells values shown, see Table S1 and the text for details [with 1 positive well the compounds itaconic acid, L‐arginine, phenyl ethylamine and 2‐hydroxy benzoic acid were also occasionally utilized]). (B) In situ temperature, stratification index (SI) and chlorophyll *a* concentration in the different months.

From each sample, 3 EcoPlates (Biolog, Inc., Hayward, USA) were inoculated with 150 μl of surface seawater per well, kept in the dark and placed within different incubation chambers: one regulated at the in situ temperature, a second one at 3°C below and a third one at 3°C above the in situ values, so that we could directly test the role of temperature on functional diversity. The choice of this temperature range was done according to ocean warming IPCC predictions for the 21st century, which estimate an increase in temperature between 1.1 and 6.4°C (Collins et al., 2013). The 6°C difference around ambient values also allowed to maximize the chances of detecting a response to temperature (i.e. too small a temperature range may have resulted in non‐significant results) while minimizing artefacts (i.e. too much warming may have induced thermal stress). Each Biolog EcoPlate™ contains 31 carbon sources in total and a water blank, all in triplicates. The presence of the redox tetrazolium violet compound in the wells indicates changes in oxidation of the substrates by developing a purple colour. The analysis of compound utilization was carried out by visual inspection of changes in colour in the plates over several weeks (up to 3–4 weeks) until we did not observe further increases in the number of positive results. Positive wells were recorded at the end of the incubation period. While in Figure 1, the utilization patterns of compounds that were utilized in at least two of the three replicate wells are represented, in the other figures and throughout the rest of the manuscript, we used the less conservative criterion of considering positive substrates when at least one of the wells showed colour formation, following Sala et al. (2008). It should be noted that strong significant relationships (*r* = 0.81–0.96, *p* < 0.05, *n* = 12) with similar slopes were consistently observed between the functional richness values using different criteria for considering positive values (change in colour in one, two or the three replicate wells).

The clustering of functional richness shown in Figure 1 was constructed applying the average linkage algorithm and Euclidean distance using the R software. To visualize potential changes in community composition between months, we used the heatmap.2 function in R for sample clustering and heatmap construction of the amplicon sequencing data described by Alonso‐Sáez et al. (2015).

## RESULTS AND DISCUSSION

Along the full year surveyed, bacterioplankton communities were able to utilize virtually all of the array of compounds present in the Biolog EcoPlate™ (30 out of the 31 compounds tested with at least 1 positive well, Table S1). Only one compound (gamma hydroxybutyric acid) was never degraded and three more (glucose‐1‐phosphate, l‐phenylalanine and putrescine) were consumed in less than 3 months in total during 2012. The clustering based on the patterns of compound utilization revealed two clearly differentiated groups roughly equivalent to consecutive periods along the annual cycle. The first group included samples from spring to early autumn (April to October), which were generally characterized by in situ temperatures above 14°C, water column stratification (SI > 6 × 10^−3^ m^−1^) and relatively low concentration of chlorophyll *a* (typically <1 μg L^−1^, including post‐bloom or non‐bloom conditions, Figure 1). The second group included mostly samples from autumn and winter (November through March), which was characterized by mixed water column conditions (SI < 4 × 10^−3^ m^−1^) and in situ temperature generally around 13°C (except in November, with 16.3°C, Figure 1). [Corrections added on 29 October 2022, after first online publication: Figure 1 citation has been added in this version.] Notably, the sample collected in May, taken during post‐bloom conditions, also clustered in this second group. It should be noted that, even if the samples analysed here followed the typical seasonal succession in bacterial community composition at the site of study (Alonso‐Sáez et al., 2015; Figure S1), the May sample was characterized by important changes in its taxonomic composition and an unusually low bacterial abundance (annual minimum of <2 × 10^5^ cells ml^−1^) (Alonso‐Sáez et al., 2015; Arandia‐Gorostidi et al., 2017). While it is clear that the bacteria actually growing in the Biolog EcoPlate™ represent only a small fraction of the whole community (Preston‐Mafham et al., 2002; Smalla et al., 1998), such marked changes in the abundance and diversity of the bacterial inoculum in May may have had an impact on the patterns of substrate utilization (Sofo & Ricciuti, 2019).

The clear seasonal structuring of functional diversity patterns agrees with previous marine (Sala et al., 2006, 2008) and freshwater (Yang et al., 2015) studies. In a study carried out in the NW Mediterranean (Blanes Bay), two seasonally differentiated clusters, which differed significantly also in nitrate and chlorophyll *a* concentration, also showed differences in the utilization patterns of 11 Biolog EcoPlate™ substrates (Boras et al., 2015). Similarly, Grover and Chrzanowski (2000) found seasonal trends in the functional diversity in four lakes: a higher response to amino and carboxylic acids in colder seasons and strong relative responses to carbohydrates in warm seasons, which they related to the seasonality of phytoplankton. The separation into two clusters based on substrate utilization patterns of Figure 1 matched exactly the periods of phytoplankton net growth (November–March, 0.22—0.98 d^−1^) and mortality (April–October, −0.20—−1.23 d^−1^) observed in parallel short‐term (4–7 days) experimental incubations of the whole microbial community (Morán et al., 2018). Figure 1 sample clustering did not mirror changes in bulk community composition (Figure S1), reinforcing the view that only subsets of the bacterial communities, those able to grow on the carbon substrates present in the EcoPlate™ wells, were responsible for the patterns observed.

In the warm, nutrient‐deficient months characterized by phytoplankton decay (Table 1), not only carbohydrates but a higher number of polymers and aminoacids were degraded (Figure 1). In this period, more than 20 substrates, up to a maximum of 26, were utilized at in situ temperature. This number decreased at lower temperatures, as the system became more eutrophic and phytoplankton switched to positive net growth. Indeed, we found a strong correlation between in situ temperature at the study site and functional richness (*r* = 0.79, *p* = 0.0022, *n* = 12). Similarly to the pattern shown by our dataset (Figure 1), the total number of utilized substrates was also lower in winter (18) than in the rest of the year at Blanes Bay in the NW Mediterranean (24–27; Sala et al., 2006). Interestingly, the number of polysaccharides hydrolyzed has been shown to decrease in high‐latitude regions (Arnosti et al., 2011; Balmonte et al., 2018) and with depth in the open ocean (Balmonte et al., 2018; Hoarfrost & Arnosti, 2017), also pointing to a potential link between temperature and functional richness. However, several environmental factors covariate with temperature both temporally (along the annual cycle) and spatially (along the vertical profile), and therefore it is uncertain if the above correlation was driven primarily by temperature. For example, in our dataset, functional richness also was negatively related with phytoplankton growth rates (# substrates = 19.08–5.62 × Phyto μ, *r* ^2^ = 0.69, *p* = 0.0009, *n* = 12).

**TABLE 1:** Mean ± SE of stratification index (SI), surface nitrate and phosphate concentrations, chlorophyll *a* concentration (Chl *a*) and net growth rate (Phyto μ), heterotrophic bacteria total abundance, contribution of HNA cells (%HNA) and net growth rate (Bacteria μ) in cool and warm conditions at the study site using 16°C as a threshold.

Temperature	SI (× 10^−3^) (kg m^−3^ m^−1^)	Nitrate (μmol L^−1^)	Phosphate (μmol L^−1^)	Chl *a* (μg L^−1^)	Phyto μ (d^−1^)	Bacteria (×10^5^ cells ml^−1^)	%HNA (%)	Bacteria μ (d^−1^)
Cool: 13.3 ± 0.3°C (6)	2.9 ± 1.3*	2.58 ± 0.67*	0.16 ± 0.03*	1.02 ± 0.24	0.25 ± 0.20*	5.90 ± 1.06	69 ± 9*	0.73 ± 0.19^a^
Warm: 18.6 ± 0.9°C (6)	13.7 ± 4.2	0.38 ± 0.09	0.05 ± 0.01	0.66 ± 0.17	−0.55 ± 0.22	6.96 ± 0.73	39 ± 4	0.32 ± 0.04

*Note*: Number of months (*n*) indicated in brackets.

* Significant differences between cool and warm conditions are indicated by asterisks (*t*‐tests, *p* < 0.05).

^a^ *t*‐test; *p* = 0.08.

**FIGURE 2:**
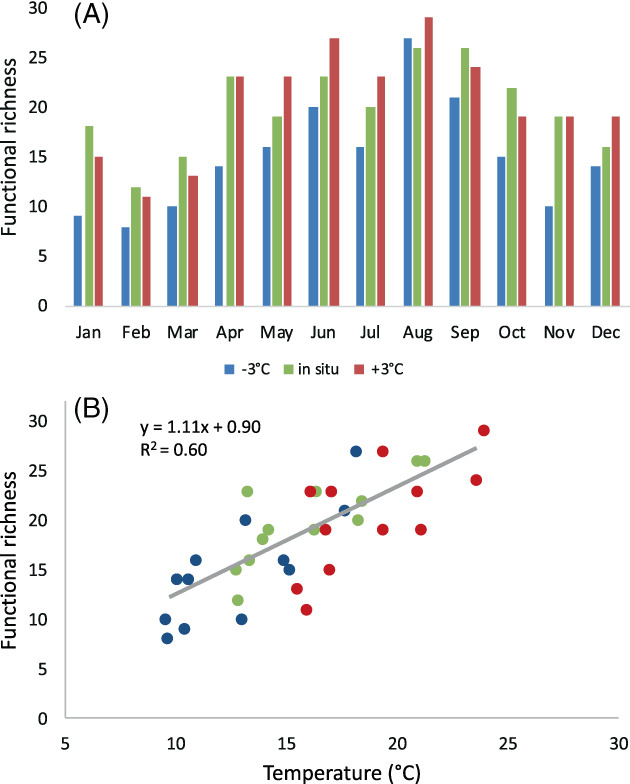
**(**A) Monthly variation in functional richness (i.e. number of individual C substrates utilized) at each incubation temperature during 2012. **(**B) Relationship between the functional richness values shown in A and the incubation temperature (*n* = 36). Fitted line is the ordinary least squares linear regression. [Corrections added on 29 October 2022, after first online publication: The final sentence of Figure 2 legend has been added in this version.]

The purpose of the 12 incubation experiments spanning 6°C of temperature around the in situ value was to experimentally test the impact of warming on carbon utilization by the same microbial community. Figure 2A shows the monthly variations of functional richness at each experimental temperature, which were consistently higher at warmer values. With all data pooled, the incubation temperature was strongly correlated with functional richness (*r* = 0.77, *p* < 0.0001, *n* = 36; Figure 2B). Earlier studies using Biolog EcoPlate™ in polar soil bacteria had found contrasting effects of temperature on functional richness (Pessi et al., 2012), while enhancements have been reported in freshwater ecosystems (Christian & Lind, 2006; Dickerson & Williams, 2014; Ylla et al., 2014). Polymers were also preferentially degraded in the warmer treatment (3°C above in situ values) in a stream biofilm (Ylla et al., 2014). Our results indicate that temperature changed the pattern of substrate utilization by including a higher diversity of compounds, in agreement with Christian and Lind (2006). The increase in bulk bacterial growth rates at higher temperatures, found in parallel incubations (Morán et al., 2018), in turn agrees with the assumption that warming enhances the rate of substrate utilization (Grover & Chrzanowski, 2000).

The slope between the individual functional richness and the three experimental temperatures shown in Figure 2A was positive in all monthly experiments, ranging from 0.3 (August) to 1.5 (April) more substrates utilized per °C increase (mean 0.9 ± 0.4 # substrates °C^−1^). This result indicates that warming activated the use of new compounds year‐round. One possible explanation is that dormant bacteria able to utilize some of the substrates as a single C source for growth would become activated (i.e. their metabolic rates would raise) by the increase in temperature, reaching a minimum abundance threshold that produced a detectable signal (Braun et al., 2006). However, that the community composition was significantly altered solely by warming seems unlikely, as demonstrated by Huete‐Stauffer et al. (2016) in parallel experiments. Alternatively, temperature activation of the metabolism of already abundant phylotypes may enhance their ability to exploit new carbon sources, especially under nutrient sufficiency (i.e. the cool period). Table 1 summarizes the environmental and heterotrophic bacteria conditions in the cool and warm periods of 2012. Although the mean bacterial abundance in the inocula did not differ between these two periods, the contribution of the copiotrophic high nucleic acid content (HNA) bacteria (Huete‐Stauffer et al., 2015; Morán et al., 2015) was significantly higher at temperatures below 16°C, resulting in more than double mean specific growth rates (0.73 vs. 0.32 d^−1^, Table 1). Copiotrophic HNA bacteria should have a higher capacity to respond to strong increases in the availability of C sources, as it takes place in the Biolog EcoPlate™, and thus we hypothesize that the temperature activation of these taxa yielded a stronger response. In this regard, Roseobacter, a typically generalist group associated to spring phytoplankton blooms, showed the highest activation energies (a measurement of the strength of the growth response to temperature) year‐round (Arandia‐Gorostidi et al., 2017).

**FIGURE 3:**
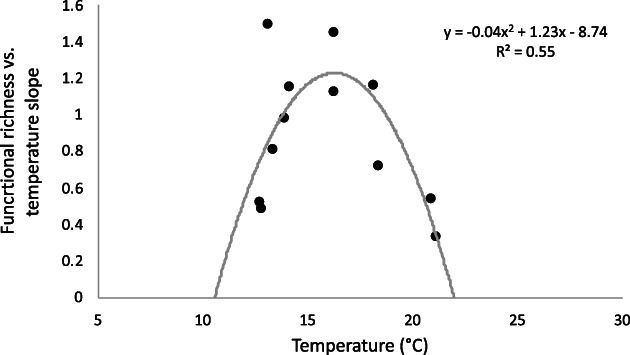
Variability of the functional richness versus temperature slope (i.e. number of substrates per °C increase) relative to in situ temperature at the study site. Fitted line is a quadratic polynomial regression. [Corrections added on 29 October 2022, after first online publication: The final sentence of Figure 3 legend has been added in this version.]

Although fewer carbon sources were utilized in situ at colder temperatures (Figure 2), the number of them being added per each °C increase in the first 4 months of the year (average temperature of 13.2 ± 0.2°C) was 64% higher than between August and October (average temperature of 20.1 ± 1.5°C). In other words, the response to experimental warming was highest during the cool period and lowest during the nutrient‐deficient conditions of the warm period (Table 1). Accordingly, the slope of increase in substrates used per °C increase followed a unimodal relationship with in situ temperature values, so that the relationship between functional richness and temperature became tighter as coastal waters warmed up to 16°C and then decreased again steadily at higher temperatures **(**Figure 3). Interestingly, the value of 16°C has been identified before as a threshold for nutrient status in this highly predictable temperate coastal ecosystem of the Southern Bay of Biscay (Morán et al., 2010, 2018): cooler temperatures are associated with nutrient‐sufficiency in a well‐mixed water column, while warmer values are typically indicative of nutrient limitation due to stratification (Calvo‐Díaz & Morán, 2006). The activation energies of bacterial specific growth rates, which is equivalent to their temperature dependence in the metabolic theory of ecology framework (Brown et al., 2004), were also significantly higher in cooler than in warmer temperatures using 16°C as a threshold, both here (Huete‐Stauffer et al., 2015; Morán et al., 2018) and in the Adriatic (Šolić et al., 2017). The different behaviour in cool (i.e. increasing slope) and warm (i.e. decreasing slope) conditions (Figure 3) is also in agreement with the growth response of other components of the microbial food web, namely primary producers and protistan grazers (Morán et al., 2018).

In conclusion, we provide evidence of a positive impact of temperature on the number of carbon substrates utilized by coastal heterotrophic bacteria, while pointing out the role of bottom‐up factors in shaping functional richness in temperate waters. In winter–spring communities, with a higher proportion of copiotrophic taxa (Alonso‐Sáez et al., 2015), temperature enhances the utilization of an increasing number of substrates. However, the temperature enhancement of functional richness rapidly decreased under nutrient‐deficient conditions in summer. Overall, this study suggests that future warming will likely result in a larger number of carbon substrates capable of being metabolized by heterotrophic bacteria, at least during nutrient‐sufficient conditions. We also suggest that the balance between production and consumption of the vast DOM pool will be differently impacted in cold (polar to temperate) and warm (subtropical to tropical) marine ecosystems.

## CONFLICT OF INTEREST

The authors do not have a conflict of interest to declare.

## Supporting information


**Appendix S1** Supporting Information

**Table S1** Functional richness (i.e., number of individual C substrates utilized) in the different monthly samples from the study site and temperature treatments, separated by the number of positive wells (*n* = 3).
**Figure S1.** Heatmap showing relative abundance of the top 25 most abundant bacterial orders in chronological order from January 2012 to December 2012. The microbial order hierarchical clustering was built according to their relative abundance. The distances between the clusters were calculated using the Lance–Williams dissimilarity function in R.

## Data Availability

Data are available upon request.
