# Supplementary material for: Temperature enhances the functional diversity of dissolved organic matter utilization by coastal marine bacteria
Source: Environ Microbiol Rep. 2022 Sep 14;15(1):31–7. doi: 10.1111/1758-2229.13123 (PMC10103754; doi:10.1111/1758-2229.13123)
Supplement: Supplementary file 2 — Table S1 Functional richness (i.e., number of individual C substrates utilized) in the different monthly samples from the study site and temperature treatments, separated by the number of positive wells (n = 3). Figure S1. Heatmap showing relative abundance of the top 25 most abundant bacterial orders in chronological order from January 2012 to December 2012. The microbial order hierarchical clustering was built according to their relative abundance. The distances between the clusters were calculated using the Lance–Williams dissimilarity function in R. [file EMI4-15-31-s002.docx]

**Supporting Information**

*Environmental Microbiology Reports (EMIR 2021-1664)*

v2. 03 February 2022

**Temperature enhances the functional diversity of DOM utilization by coastal marine bacteria**

Xosé Anxelu G. Morán^1*^, Nestor Arandia-Gorostidi^1,2^, Tamara Megan Huete-Stauffer^1^, Laura Alonso-Sáez^1,3*^

^1^ Centro Oceanográfico de Gijón/Xixón, IEO, Gijón/Xixón, Spain

^2^ Department of Earth System Science, Stanford University, Stanford, USA.

^3^AZTI, Marine Research, Basque Research and Technology Alliance (BRTA), Sukarrieta, Spain

*These authors contributed equally to this work

Corresponding authors: [xelu.moran@ieo.es](mailto:xelu.moran@ieo.es), [laura@azti.es](mailto:laura@azti.es)

*Running title*: Temperature impact on bacterial functional diversity

**Table S1.** Functional richness (i.e., number of individual C substrates utilized) in the different monthly samples from the study site and temperature treatments, separated by the number of positive wells (n=3).

| **Date** | **Treatment** | **Temperature** | **1 positive** | **2 positives** | **3 positives** |
| --- | --- | --- | --- | --- | --- |
| 18-Jan-12 | -3°C | 13.9 |  |  |  |
|  | in situ | 10.4 |  |  |  |
|  | +3°C | 16.9 |  |  |  |
| 10-Feb-12 | -3°C | 12.8 |  |  |  |
|  | in situ | 9.6 |  |  |  |
|  | +3°C | 15.9 |  |  |  |
| 13-Mar-12 | -3°C | 12.7 |  |  |  |
|  | in situ | 9.5 |  |  |  |
|  | +3°C | 15.5 |  |  |  |
| 2-May-12 | -3°C | 13.2 |  |  |  |
|  | in situ | 10.0 |  |  |  |
|  | +3°C | 16.1 |  |  |  |
| 23-May-12 | -3°C | 14.2 |  |  |  |
|  | in situ | 10.9 |  |  |  |
|  | +3°C | 17.0 |  |  |  |
| 14-Jun-12 | -3°C | 16.3 |  |  |  |
|  | in situ | 13.1 |  |  |  |
|  | +3°C | 19.3 |  |  |  |
| 3-Jul-12 | -3°C | 18.2 |  |  |  |
|  | in situ | 14.9 |  |  |  |
|  | +3°C | 20.9 |  |  |  |
| 2-Aug-12 | -3°C | 21.2 |  |  |  |
|  | in situ | 18.1 |  |  |  |
|  | +3°C | 23.9 |  |  |  |
| 11-Sep-12 | -3°C | 20.9 |  |  |  |
|  | in situ | 17.6 |  |  |  |
|  | +3°C | 23.6 |  |  |  |
| 19-Oct-12 | -3°C | 18.4 |  |  |  |
|  | in situ | 15.1 |  |  |  |
|  | +3°C | 21.1 |  |  |  |
| 15-Nov-12 | -3°C | 16.2 |  |  |  |
|  | in situ | 13.0 |  |  |  |
|  | +3°C | 19.3 |  |  |  |
| 11-Dec-12 | -3°C | 13.3 |  |  |  |
|  | in situ | 10.6 |  |  |  |
|  | +3°C | 16.8 |  |  |  |
|  | in situ | 10.6 | 5.90 ± 1.06 | 69 ± 9* | 0.73 ± 0.19^a^ |
|  | +3°C | 16.8 | 6.96 ± 0.73 | 39 ± 4 | 0.32 ± 0.04 |

**
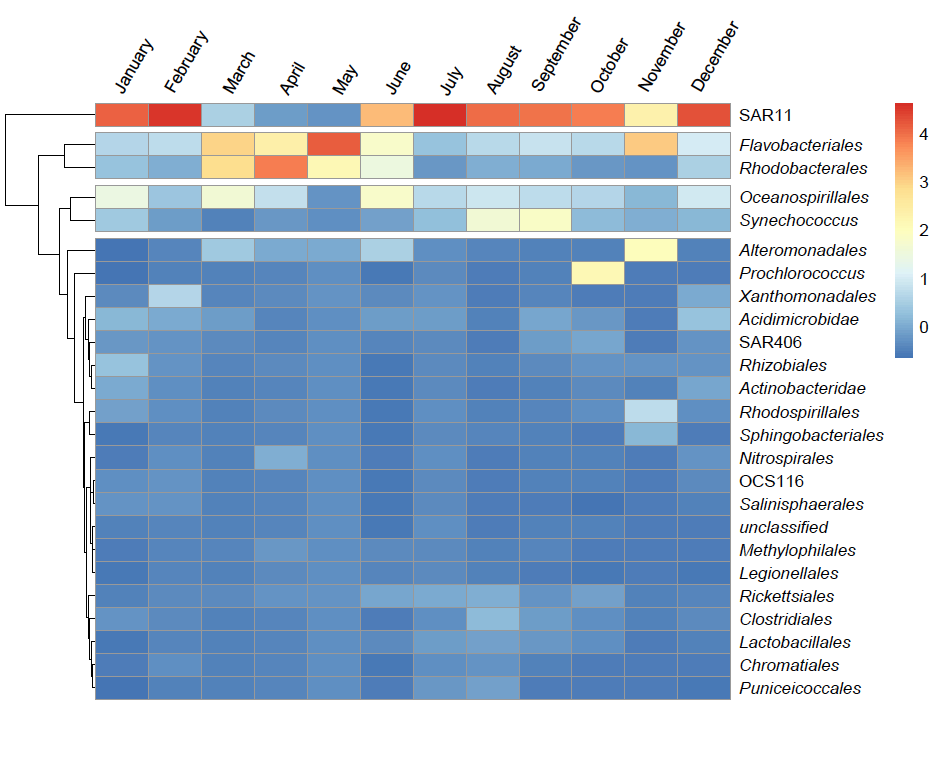
Fig. S1.** Heatmap showing relative abundance of the top 25 most abundant bacterial orders in chronological order from January 2012 to December 2012. The microbial order hierarchical clustering was built according to their relative abundance. The distances between the clusters were calculated using the Lance–Williams dissimilarity function in R.
